# The KM-Algorithm Identifies Regulated Genes in Time Series Expression Data

**DOI:** 10.1155/2009/284251

**Published:** 2009-10-07

**Authors:** Martina Bremer, R. W. Doerge

**Affiliations:** ^1^Department of Mathematics, San Jose State University, One Washington Square, San Jose, CA 95192, USA; ^2^Department of Statistics, Purdue University, 150 N. University Street, West Lafayette, IN 47907, USA

## Abstract

We present a statistical method to rank observed genes in gene expression time series experiments according to their degree of regulation in a biological process. The ranking may be used to focus on specific genes or to select meaningful subsets of genes from which gene regulatory networks can be built. Our approach is based on a state space model that incorporates hidden regulators of gene expression. Kalman (K) smoothing and maximum (M) likelihood estimation techniques are used to derive optimal estimates of the model parameters upon which a proposed regulation criterion is based. The statistical power of the proposed algorithm is investigated, and a real data set is analyzed for the purpose of identifying regulated genes in time dependent gene expression data. This statistical approach supports the concept that meaningful biological conclusions can be drawn from gene expression time series experiments by focusing on strong regulation rather than large expression values.

## 1. Introduction

Novel gene expression technologies (e.g., microarrays, next-generation sequencing, etc.) make it possible to study the simultaneous expression of an ever increasing number of genes [1, 2]. As these technologies become more available and affordable, the size and complexity of gene expression experiments will continue to increase. In time series studies, for example, microarray-based gene transcript measurements have historically been recorded at several time points over the course of a biological process (e.g., development, response, etc.). Well-known examples of microarray time series experiments include studies on the yeast cell cycle [3], the reaction of mice to acute corneal trauma [4], the life-cycle of drosophila [5], and embryonal development of the rat nervous system [6]. In most time series gene expression experiments “treated” samples are compared to a zero time point “reference” or “control” sample, and a (log-fold) change or difference is calculated for each gene at each time point under both equally spaced or unequally spaced conditions. While in the past the number of time points under investigation was usually limited to fewer than ten, the current progression is toward extensive studies that include well beyond 50 time points [5]. Furthermore, with the rising popularity of next generation sequencing (e.g., Solexa) as applied to gene expression studies we anticipate that the size and nature of gene expression experiments will both continue to grow and to provide challenging statistical issues that need to be addressed. Here, we present a statistical method that ranks genes via their expression as gained from microarray-based time series experiments and in accordance with their degree of regulation in a biological process. Although the statistical approach is presented using microarray technology, the method is independent of the technology that presents the data and provides meaningful biological conclusions that are based on regulation rather than expression.

To date a variety of statistical methods have been developed for the analysis of time series microarray data, and even fewer for next generation sequencing. Clustering methods have been used extensively to deduce the function of previously unknown genes by comparing their expression profile to known genes in the same cluster [7] even though it is well known that similar expression profiles do not necessarily imply the same biological function [8]. Statistical approaches that describe gene expression as a complex (noisy) function of internal and external cell stimuli have also been implemented. These approaches attempt to construct gene regulatory networks based on Boolean models [9–11], vector autoregressive models [12], empirical Bayesian methods [13], or Bayesian networks [14, 15] (e.g., state space models) and are often thought of as directed graphs, in which the observed genes and hidden regulators are considered as nodes and the arrows between nodes as causal (temporal) relationships. In gene expression (microarray) applications the advantage of state space models is that they are easily extendable to the case of unequally spaced time points and they allow the inclusion of unobservable regulators in the model. In fact, these hidden regulators may include, but are not limited to, genes that were not apriori of interest in the experiment (e.g., transcription factors). A disadvantage of state space models is their poor identifiability, especially in high-dimensional data. In fact, in previous attempts to analyze gene regulation networks with state space models [16, 17], strong restrictions had to be placed on the model parameters.

Most of the existing analytic methods for discovery of gene regulatory networks rely on the preselection of a subset of genes that are subsequently analyzed. Unfortunately, these methods are only feasible if the set of genes from which the network is built is small (i.e., magnitude of hundreds, not thousands) and is typically justified by the underlying assumption that not all of an organism's thousands of genes are involved in a specific temporal process (e.g., the cell cycle). Under such an assumption arbitrary cutoff values are used to compare a gene's absolute or relative maximum expression to a control tissue (zero time point) to determine whether it should be included or excluded from further consideration [18]. Although such a criterion is not biologically meaningful, the impact is significant and results in a large percentage of the available observations and information are excluded *a priori* from any subsequent analysis [19, 20].

Our proposed approach is called the KM-algorithm, it does not depend on any *a priori* analysis to reduce the dimension of the data, and it is based on Kalman (K) smoothing [21] and maximum (M) likelihood estimation. It is a modified EM-algorithm [22], in which the conditional expectation step (E-step) is replaced by a Kalman smoothing procedure (K-step) that estimates the hidden regulators. In the maximization step (M-step) the model parameters are updated through a gradient ascend procedure that increases the model likelihood for the given set of observations while simultaneously assuring validity of the model. The KM-algorithm is numerically and computationally feasible for application to gene expression data consisting of thousands of gene observations. When whole genome investigations force the number of observations to be extremely large (tens of thousands of genes), a partitioning method can be employed to reduce the computational load of the problem. Essentially, the partitioning method repeatedly splits the data into smaller subsets upon which the KM-algorithm operates. The results from the KM-algorithm are then combined to yield a single result for each gene. Using a novel criterion that ranks genes according to their degree of regulation (rather than their maximum expression) gives rise to a meaningful subset of genes. Since the KM-algorithm itself is based on a state space model for gene regulation, it allows the inclusion of hidden regulators. Depending on whether the hidden regulators are unobserved gene expression values or transcription factors, they can be used to model gene-gene and potentially gene-protein interactions (i.e., a protein, that results from the expression of a gene that is not on the array, regulates the expression of an observed gene).

The performance of the KM-algorithm is studied via simulation using gene expression time course data of varying size (gene number) and length (number of time points). While we have based our simulations on microarray technology, any technology can be assumed/employed. The ranking result as gained from our approach is evaluated by the position of the (simulated) regulated genes in the final list. These simulation studies provide both guidelines and recommendations for the minimum number of time point observations that a gene expression experiment should include in order to achieve a desired degree of statistical separation (i.e., accuracy) between regulated and unregulated genes.

## 2. Methods

### 2.1. State Space Model for Gene Regulation

A statistical model for any complex biological process such as gene regulation must make simplifying assumptions. The choice of a model is a compromise between flexibility of the model (being able to explain a large proportion of the observed variance) and simplicity of the model itself. In this work gene regulation is modeled through a discrete time state space model with hidden regulators and Gaussian error terms:

(1) $$\begin{array}{cc} Z_{t} & =GY_{t}+\epsilon_{t},\\ Y_{t} & =FY_{t-1}+\delta_{t}. \end{array}$$

The *Z*_*t*_ are the *n*-dimensional vectors of observed gene expression values at time points *t* = 1,…, *T*, and *Y*_*t*_ are the *m*-dimensional regulators that determine the expression of some of the observed genes. These regulators (*Y*_*t*_) do not necessarily have to be gene expression. Here, the observations are assumed to be equally spaced; however the theory can be extended to the situation of unequally spaced observations (discussed later). The error terms *ϵ*_*t*_ and *δ*_*t*_ are assumed to be mean zero multivariate Gaussian errors with covariance matrices Σ_*ϵ*_ and Σ_*δ*_, respectively. These errors are important since they model the measurement error and biological variation in the regulators, respectively. The model assumption that the error terms have mean zero requires any systematical measurement bias to be removed prior to analysis. In microarray data analysis this is usually achieved through preprocessing of the data and normalization techniques such as print-tip normalization or dye-swap normalization. Although measurement errors on gene expression are assumed to be uncorrelated, thus Σ_*ϵ*_ is a diagonal matrix, this does not mean, nor imply, that the observed gene expression values themselves are uncorrelated. In fact, the observed gene expression values are modeled as linear combinations of regulators which are themselves correlated. The system matrix *F* describes the temporal development of the regulators, and the gene regulation matrix *G* is identifiable only if the dimension *m* of the state space is smaller than the number of observed time points *T*. Typically, in biological experiments the number of relevant regulators is small, and therefore this issue is not anticipated as problematic. Something that is dealt with later, but worth noting now, is that the gene regulation matrix *G* and the system matrix *F* are not unique, and any renumbering of the hidden regulators will most likely lead to different gene regulation and system matrices. We chose this model because it provides a large degree of flexibility in the hidden regulators which could be cell internal or external components. On the other hand, the simplifying assumptions of time independent regulation matrices *G* and *F* and a linear relationship between regulating elements and gene expressions are biologically reasonable.

### 2.2. KM-Algorithm

A modified EM-algorithm is employed to estimate the parameters of the state space model. The parameters that are of interest, and that need to be estimated in the state space model (1), consist of the gene regulation matrix *G*, the system matrix *F*, the covariance matrices of the biological error Σ_*δ*_, the measurement error Σ_*ϵ*_, and the mean and covariance matrix for the Gaussian distribution of the initial regulator state *Y*_0_ ∼ *N*(*μ*, Σ).

The KM-algorithm starts with random initial values for the model parameters and then alternates between the Kalman smoothing (KS) estimates of the hidden regulators *Y*_0_,…, *Y*_*T*_ and the (restricted) maximum likelihood estimates of the model parameters. Kalman smoothing is an engineering technique that computes the conditional expectations of the hidden state variables, given the complete set of observations [21]. Since the model parameters are fixed, in practice, when the these parameters are not known, model parameters estimates are used. Computing KS-estimates of the hidden regulators first requires a forward pass through the data to compute filtering estimates and then a backward pass to obtain the smoothing estimates. Fortunately, both procedures consist mainly of matrix multiplication and addition and the inversion of one symmetric *n* × *n* and one symmetric *m* × *m* matrix, respectively. The numerical complexity of the Kalman filtering and smoothing procedure is *O*(*l* ^3^), where *l* is the number of model parameters.

To update the model parameters, the likelihood function (*L*) of the regulator values given the complete set of observations is maximized

(2) $$\begin{array}{c} L_{F,G,\Sigma_{\delta },\Sigma_{\epsilon },\mu ,\Sigma }\left(Y_{0},\ldots ,Y_{T}\;|\;Z_{1},\ldots ,Z_{T}\right) \end{array}$$

with respect to the model parameters. Due to numerical instability in the algorithm with regards to computation of the covariance matrix Σ_*δ*_, the value of Σ_*δ*_ that maximizes the likelihood function is not necessarily positive definite. Since a positive definite covariance matrix is required in the subsequent Kalman smoothing step, the algorithm is amended with a Cholesky square-root decomposition of the covariance matrix with a subsequent gradient ascent for the likelihood function [23]. The algorithm is terminated if the proportional increase in the model log-likelihood falls under a threshold (e.g., ≤0.05%).

### 2.3. The Regulation Criterion

Based on the state space model (1) for gene regulation, we formulate and propose a criterion that allows the identification of regulated genes in a particular process of interest. The formulation of the criterion is independent of the numbering of the hidden regulators. Therefore, several model estimates resulting from repeated applications of the KM-algorithm with different initial values may be averaged which effectively provides a more powerful gene ranking process.

In the state space model (1) the gene regulation matrix *G* has zeroes in the rows that correspond to unregulated genes. Large positive or negative entries in a gene's *G*-row indicate that the gene is up- or downregulated by the corresponding regulator. The sum of squared estimated *G* row entries can therefore be used in a criterion for gene regulation. Because genes with greater variation in expression over time also tend to have larger gene regulation matrix entries, the sum of squared estimated *G* row entries ${\hat{g}}_{ij}$ is standardized by the temporal variance (*V*_*i*_) of each gene:

(3) $$\begin{array}{c} R_{i}=\frac{\sum_{j=1}^{m}{\hat{g}}_{ij}^{2}}{V_{i}},\;\text{where}\;\;V_{i}=\mathrm{Var}\;\left(Z_{t}^{\left(i\right)},\;\;t=1,\ldots ,T\right). \end{array}$$

This criterion (3) provides one regulation value *R*_*i*_ for every gene in the experiment. Followup experiments and real-time PCR validation to discover regulated genes should be focused on the genes with the highest regulation criterion. If the KM-algorithm is implemented repeatedly for the same set of observations with different initial values, the resulting regulation criteria are directly comparable and may be averaged.

### 2.4. Selection of Model Dimension

Before the generalized EM-algorithm can be employed for estimation of the model parameters, the dimension of the state space must be estimated. Conventional model selection methods such as Bayes Information Criterion (BIC) [24] or Akaike Information Criterion (AIC) [25] fail in many (microarray) applications, since the number of observations (genes) and the number of model parameters to be estimated may be extremely large (in the thousands).

Since traditional model selection criteria are not well suited for this particular application, a method that is based on the autocovariances of the observed gene expression values is employed [26]. If we let *H* be the block-Hankel matrix of observation autocovariances ${\hat{\Gamma }}_{i}$ estimated from the gene expression observations *Z*_*t*_, *t* = 1,…, *T*, then

(4) $$\begin{array}{c} H=\left(\begin{array}{cccc} {\hat{\Gamma }}_{1} & {\hat{\Gamma }}_{2} & \cdots & {\hat{\Gamma }}_{p}\\ {\hat{\Gamma }}_{2} & {\hat{\Gamma }}_{3} & \cdots & {\hat{\Gamma }}_{p+1}\\ \vdots & \vdots & \ddots & \vdots \\ {\hat{\Gamma }}_{p} & {\hat{\Gamma }}_{p+1} & \cdots & {\hat{\Gamma }}_{2p-1} \end{array}\right),\;\;{\hat{\Gamma }}_{i}=\frac{1}{T}\overset{T-i}{\underset{t=1}{\sum }}Z_{t+i}Z_{t}^{\prime }. \end{array}$$

In this setting *p* is the maximum biologically relevant time-lag between a gene and its regulator. That is, a gene or regulator may influence the expression of another gene at most *p* experimental time units in the future. Usually time series expression (microarray) experiments are designed so the maximum relevant time-lag is moderate (*p* = 1, 2, 3). In the absence of error, the rank of the matrix *H* equals the number of states required to characterize the observations *Z*_*t*_ in the state space model (1).

In practice the observed gene expression values, *Z*_*t*_, are subject to both biological and technical measurement errors, and the rank of *H* cannot be used directly to choose the state space dimension *m*. However, a singular value decomposition (SVD) of *H* can be performed, and the number of singular values of comparably large magnitude is used as an estimate for the most appropriate state space dimension *m*. Specifically, the singular values of the estimated Hankel-autocovariance matrix are computed and standardized to a 0–1 scale. The number of singular values of magnitude ≥0.8 is used as an estimate for the state space dimension. An arbitrary threshold of 0.80 is chosen based on our experience that the number of singular values of this magnitude is representative for the state space dimension. Numerical computation of singular values is not time extensive but requires a large amount of available memory (e.g., 1.6 GB for observations on *n* = 2000 genes and *p* = 3). Singular value decomposition of the Hankel matrix in general has complexity *O*((*np*)^3^). However, since the matrix has rank strictly less than *T*, we only need to compute the *T* − 1 largest eigenvalues. Efficient SVD algorithms, specifically for symmetric matrices, can be employed for this task [27].

A further advantage of using a method based on the autocovariances of the observed gene expressions rather than conventional model selection procedures is that it does not require fitting many models of different dimensions. Instead, the estimated autocovariances of the observed variables are computed from the observations directly. Since the model fitting step is computationally much more time extensive than the singular value decomposition of *H*, this allows for a significant reduction in overall computation time.

### 2.5. Unequally Spaced Observations

In the state space model (1) the gene observations are assumed to be equally spaced in time. In most practical experiments, however, the time steps or intervals between observations are not equal. Let Δ*t* be largest common factor of the intervals between measurements. Specifically, every time step can be expressed as an integer multiple of Δ*t* such that the state space model in (1) is modified:

(5) $$\begin{array}{cc} Z_{t_{k}} & =GY_{t_{k}}+\epsilon_{t_{k}},\\ Y_{t_{k}} & =F^{j_{k}}Y_{t_{k}-1}+\delta_{t_{k}}, \end{array}\;k=1,\ldots ,T,$$

where *j*_*k*_ = (*t*_*k*_ − *t*_*k*−1_)/Δ*t* is an integer for *k* = 1,…, *T*. For unequally spaced observations, the KS estimates of the hidden regulators can be obtained in an similar manner to the equidistant time point case, where the system matrix *F* is replaced with the appropriate *F* ^*j*_*k*_^ in both the filtering and smoothing recursion. However, the covariance matrix *τ*_*j*_*k*__Σ_*δ*_ of the error terms *δ*_*t*_*k*__ will depend on the spacing *j*_*k*_ between observations. Icaza and Jones [28] show how to apply the Kalman filtering and smoothing procedure to the case of multivariate observations that are unequally spaced in time. The terms in the conditional likelihood function that depend on the system matrix *F* will no longer be quadratic in *F* (as is the case in model (1)). They can be represented as a polynomial expression in *F*. The solution to the matrix equation

(6) $$\frac{\partial }{\partial F}L_{F,G,\Sigma_{\delta },\Sigma_{\epsilon },\mu ,\Sigma }\left(Y_{0},\ldots ,T_{T}\;|\;Z_{1},\ldots ,Z_{T}\right)=0$$

may not exist in closed form but can be obtained numerically for instance through a gradient ascent.

### 2.6. Partitioning Method for Large Data Sets

Many gene expression (time series) data sets have tens of thousands of observations. For example, the Arabidopsis ATH1 Affymetrix microarray represents more than 24000 genes. Since the numerical expense of estimating the parameters of a state space model increases quadratically in the number of observed genes, parameter estimation for the complete set of (genes) observations quickly becomes computationally challenging. To address this challenge without restricting the gene space, or limiting the KM-algorithm, one can randomly partition the data into several smaller subsets of approximately equal size. The KM-algorithm can then be implemented, with different initial starting values, repeatedly for each subset. When sufficient computing capacity is available, these calculations can be carried out in parallel. The regulation criterion results for genes from all subsets are collected, and the procedure is repeated with a different random partitioning of the data. The results from the KM-algorithm are then combined via averaging to yield a single result for each gene. Biologically, splitting the observed data into subsets has no ill effect if all regulators are unobserved components, such as protein levels. However, if some of the regulators are observed gene expression values themselves, then splitting the data set may potentially ignore any gene-gene interactions. Therefore, the random partitioning is repeated with different subsets, to accommodate possible gene-gene interactions.

## 3. Results

### 3.1. Simulated Data

Data of different sizes (gene numbers *n* = 500, 1000, 2000) and lengths (time points *T* = 20, 40, 100) are simulated. For comparability, in each case *m* = 10 regulators are simulated with the same system matrix *F*. The regulators are autoregressive processes (AR(*p*)) of maximum order *p* = 3. They are allowed to differ in their autocorrelation and temporal variance to reflect a range of different biological applications. In each data set, twenty genes are simulated as regulated. Effectively, the *G*-row entries corresponding to these genes contain nonzero entries. The temporal variances of the unregulated genes (diagonal entries of Σ_*ϵ*_) are chosen to cover the range of temporal variances of the regulated genes.

### 3.2. Evaluating the Ranking of Genes

For simulated data where some of the genes are simulated or known as regulated, performance of the KM-algorithm is evaluated by ranking the genes. A perfect ranking result is one in which the regulation criterion values of the regulated genes surpass those of all unregulated genes. Due to both technical and biological variation in the observations this is rarely the case, and therefore an objective measure that describes the “goodness of ranking” of the KM-results is required. The goodness of ranking (GR) measure used here is based on the average ranking positions of the regulated genes. It assigns a value of one to a perfect ranking and a value of zero to the average random ranking of regulated and unregulated genes. Note that negative GR-measure values are possible and will occur if the regulated genes are listed at the bottom of the ranking list.

### 3.3. Simulation Example

The KM-algorithm is applied to a simulated data set with *n* = 1000 genes, twenty of which are regulated. All genes are observed at *T* = 100 equally spaced time points. Applications of the KM-algorithm are based on five different sets of initial starting values. Each time the regulation criterion is computed, the five values for a gene are averaged.

**Figure 1:**
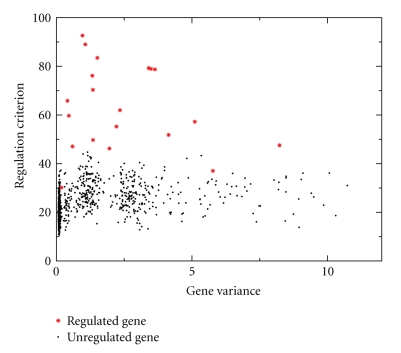
Averaged regulation criterion for five applications of the KM-algorithm as applied to simulated data with *n* = 1000 genes at *T* = 100 time points. Regulated genes are plotted as red stars and unregulated genes as black dots.

In Figure 1 the calculated regulation criterion values are plotted against the temporal variances (*V*_*i*_) of the genes. Historically, the temporal variances are typically used as an indicator for selection of genes in microarray time series analysis [7]. As expected, the ranking result is not perfect, and some unregulated genes have larger regulation criterion values than regulated genes. In fact, the GR-measure for the ranking outcome in this simulated example is 0.7565. However, when compared to the traditional method for selecting genes based on the temporal variances, selection of genes using a high regulation criterion values rather than a high temporal variation yields a considerably larger percentage of genes correctly identified as regulated in the chosen subset. Recall that twenty of the simulated 1000 genes in this example are regulated, and the remaining 980 simulated genes are unregulated. Table 1 summarizes the percentages of correctly identified genes when selecting the top 1%, 5%, 10%, or 20% of genes as regulated according to temporal variance or the proposed regulation criterion.

### 3.4. Power Study

A major advantage of the regulation criterion that is applied here is that it is independent of the order of the unobservable regulators. Hence, the results from two or more applications of the KM-algorithm on the same data set with different initial starting values may be averaged to yield higher power in detecting regulated genes.

**Figure 2:**
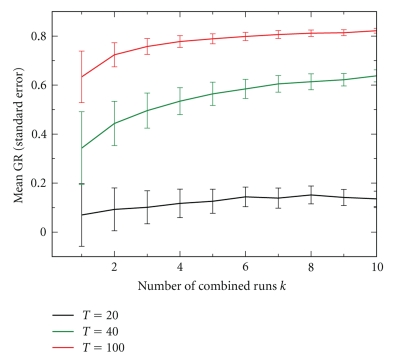
Average goodness of ranking (GR) measure (with standard error) for averaging *k* (*k* = 1,…, 10) regulation results obtained through repeated application of the KM-algorithm with different initial starting values.

Figure 2 demonstrates that a balance can be achieved between increased power and increased computation expense. The KM-algorithm is applied 1000 times each to three different simulated data sets with observations on *n* = 1000 genes at *T* = 20, 40, and 100 time point observations, respectively. For each data set the 1000 regulation criterion results are grouped into subsets of size *k* (*k* = 1,…, 10) and averaged. For the averaged regulation criterion results, the GR-measure of ranking quality is computed. The average GR-measure values together with their respective standard errors are reported in Figure 2. Clearly, averaging more implementations of the KM-algorithm improves the quality of the final ranking result. However, while the improvement is drastic for smaller values of *k*, a large number of implementations may not be worth the added computational expense. As can be seen, a good tradeoff between improvement in gene ranking and computation time appear to be values around *k* = 5 implementations of the KM-algorithm.

Figure 2 also clearly illustrates that including more time points in the design of an experiment vastly improves the gene ranking results. In the case of *T* = 20 time points, the GR-measure values are only slightly better than those that would be achieved by a random ranking of all the observed genes (GR = 0). However, for *T* = 40 time points, the results are drastically better. A perfect gene ranking (GR = 1) cannot be expected even for very long time series, due to both the technical error in the observations and biological variation in the regulators. Based on these simulations, a minimum length of *T* = 40 time point observations is recommended for most microarray or expression time series experiments. 

### 3.5. Selection of Model Dimension

**Figure 3:**
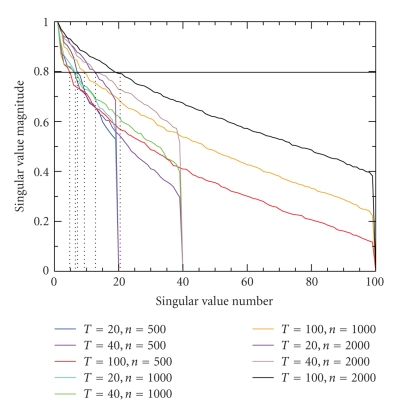
Standardized singular values of the block Hankel matrix of autocorrelations for maximum biological time lag *p* = 1.

A common difficulty in many complex statistical models is selecting the appropriate model dimension. Because of the vast number of genes, microarray applications are especially challenging when selecting an appropriate model. The model selection method presented earlier is based on the autocovariances of the (gene) observations. For nine simulated data sets of different sizes and lengths the block Hankel matrix of estimated autocovariances is computed for maximum biological time lag *p* = 1. A singular value decomposition is performed, and the number of singular values which exceed 80% of the largest singular value is used as an estimate for the model dimension. The standardized singular values for the nine data sets are plotted in Figure 3. The true model dimension for each simulated data set is *m* = 10 (i.e., there are 10 regulators of gene expression). The estimated values (Figure 3; vertical dotted lines) as gained from model selection range from 4 to 18.

**Figure 4:**
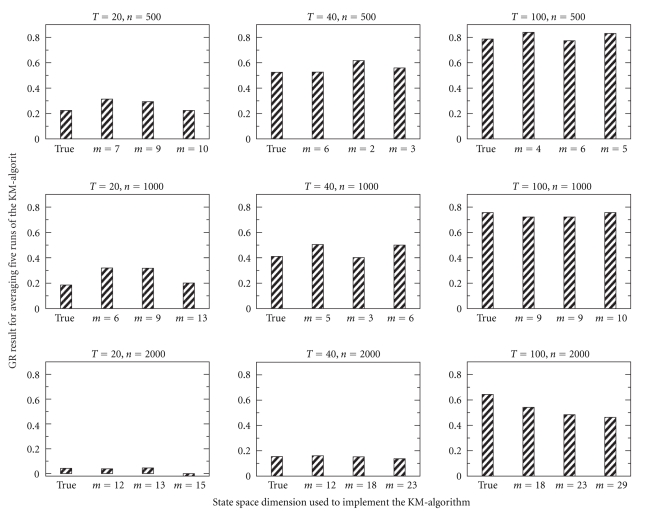
For nine simulated data sets of different size *n* and length *T*, model selection is performed with different maximum biological time lags *p* = 1, 2, 3. The results of the model selection process are noted on the *x*-axis of each data set (left bar represents true model dimension *m* = 10, right three bars represent *p* = 1, 2, 3 in that order). At the true model dimension *m* = 10 and the selected dimensions the KM-algorithm is applied five times and regulation results averaged. The “goodness of rank” (GR) measure of the resulting gene ranking is shown.

Because the maximum biological relevant time lag *p* is chosen by the experimenter, it is important to assess how different choices of *p* influence the model selection process. Figure 4 demonstrates the influence that a misspecification of the maximum relevant time lag *p* has on both the selected model dimension and the gene ranking result as measured by GR. For all nine simulated data sets the true dimension *m* = 10 is compared to those selected by the autocovariance based model selection method with time lags *p* = 1, 2, and 3, respectively. The true maximum time lag for the simulated data is in fact *p* = 3 as the regulators are AR(*p*) processes of maximal degree 3.

As seen in Figure 4 the gene ranking results are strongly influenced by the length of the observed time series. Observations at more time points yield improved ranking results. The size of the data set (number of simultaneously observed genes) also influences the results. Since the number of regulated genes in each data set is constant, larger numbers of observed genes give rise to weaker ranking results as the average list position of the regulated genes tends to be smaller.

A more detailed discussion of the effects of misspecification of the maximum relevant time lag *p* on the selected model dimension and subsequent ranking of genes by the KM-algorithm can be found in [29].

### 3.6. Partitioning Method

For larger microarray experiments with thousands of genes the proposed partitioning method is demonstrated. The KM-algorithm is implemented five times for each data set that consists of *n* = 2000 genes that are observed at *T* = 20, 40, and 100 time points, respectively. The ranking results are averaged, and a final gene ranking is obtained for each data set. To compare the KM-algorithm implementation on a whole data set with a partitioned data set, the same three data sets are then analyzed using the partitioning method. Specifically, each data set is partitioned randomly into four smaller subsets of 500 genes. Model selection is performed for each subset separately, and the KM-algorithm is implemented (at the selected model dimension) three times each with different initial starting values. The three resulting regulation criterion values for each gene are averaged. To capture gene-gene interactions, the random partitioning and subsequent analysis is repeated to yield a total of five mean regulation criterion estimates for each gene. These results are averaged again, to obtain one final regulation result for each of the *n* = 2000 genes in the data set.

Table 2 compares the GR measure of ranking for the results from analyzing the large data set as a whole with those obtained by utilizing the partitioning method. Specifically for shorter time series, the ranking results obtained by the partitioning method surpass those obtained by analyzing the entire data set. This can be explained by the fact that the partitioning method effectively uses 15 applications of the KM-algorithm, compared to five applications when the data set is considered in total (i.e., not partitioned). The increased power that is achieved through more applications of the KM-algorithm is offset by potentially unobserved gene-gene interactions in the partitions.

### 3.7. Yeast Cell Cycle Data

The partitioning method is applied to data generated by a well-studied yeast cell cycle experiment [3]. Since these data are so well studied by many investigators using many approaches, direct comparisons can be made between the methods and results. The yeast experiment cells from a CDC15 temperature sensitive yeast mutant were harvested every ten minutes under growth conditions at 19 equally spaced time points. At each time point 6308 distinct genes were evaluated using spotted cDNA microarrays. In the original experiment and analysis, Fourier transformation and the correlation of gene temporal profiles with those of known regulating genes were used to classify 799 genes as regulated in a cell cycle dependent manner.

The KM-algorithm in combination with the partitioning method is applied to the original Spellman log _2_ transformed yeast expression ratios. The 6308 genes are randomly partitioned into nine subsets of 630 and one subset of 638, respectively. Model selection via singular value decomposition of the autocovariance matrix is performed for each subset. Subsequently, the KM-algorithm is implemented three times for each subset at the selected model dimension with different initial values. Finally, the regulation criterion results are averaged over both repeated applications of the algorithm and repeated partitions.

**Figure 5:**
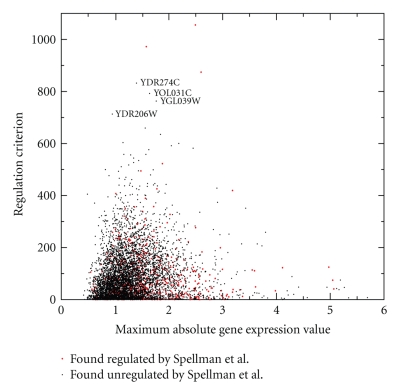
Regulation criterion values obtained by applying the partitioning method to Spellman's CDC15 yeast data plotted against the maximum absolute expression value of each gene. Genes that have been found to be cell cycle regulated by Spellman are plotted as red dots. Four genes that received high regulation criterion values, yet were not found to be cell cycle regulated by the original Spellman analysis, are labeled.

Figure 5 shows the calculated regulation criterion values plotted against the maximum absolute expression of each gene. In the original Fourier transformation-based analysis [3] genes with both small and large absolute expression were identified as cell cycle regulated. The implementation of the KM-algorithm using the same data identified the top three genes as cell cycle regulated. These same genes were also recognized by Spellman et al. as cell cycle regulated. However, the genes with the next highest regulation criterion values (i.e., * YDR274C, YOL031C, YGL039W*, and * YDR206W*) were not identified as cell cycle regulated in the original Spellman et al. results. Interestingly, in later yeast experiments * YOL031C* was found to be involved in processes during cotranslational membrane targeting [30], and * YGL039W* and * YDR206W* were found to be involved in telomeric maintenance [31, 32]. To our knowledge the molecular function of * YDR274C* remains unknown.

## 4. Conclusions

 An efficient approach to ranking genes according to their degree of regulation in the observed biological process is presented using the novel KM-algorithm. While the KM algorithm is implemented on gene expression data in a microarray setting, it is technology independent. The ranking that results from a KM analysis can be used to select genes for individual study or to fit regulatory networks with existing methods that rely on the preselection of a smaller subset of genes. The selection of genes according to regulation, rather than absolute expression or variation over time, is biologically more meaningful and has great potential to aid in the discovery of regulatory pathways and networks.

The major benefit of using a state space model in the proposed KM-algorithm is the inclusion of hidden regulators. This feature is especially important when the focus is on constructing regulatory networks, since it provides an opportunity to discover additional regulating genes that may not be in the current network. It is expected that the application of the KM-algorithm to situations where the regulators of gene expression are both known and unknown is fairly broad (e.g., transcription factors, DNA methylation, and cell external stimuli). Furthermore, since the model provides a convenient way to integrate the technical variation, that is an integral part of any technology, separately from the biological variation in the observed organisms, there is huge potential for novel discoveries.

It is not surprising that complex statistical models are required to represent both the complexity and dependence structure of gene regulatory networks. Parameter estimation for complex models with different sources of variation, and simultaneous gene observations on a large number of variables, is one of our greatest challenges. In particular, the estimation of parameters in a Bayesian network, such as a state space model, is an *np* hard problem [33]. Therefore, it is essential that data reduction occurs in a biologically meaningful way that is aimed at retaining as much relevant information about the network as possible.

The KM-algorithm, which ranks genes based upon their degree of regulation, is easy to implement, and the calculations are feasible even for very large microarray data sets. Simulations show that the quality of gene ranking for time series of medium length (*T* > 40) is good and improves as the number of time point observations increases. It is anticipated that new technologies which are less expensive and include flexible design (e.g., CombiMatrix; [2]) will give rise to experiments with increased time point observations. The affect of using a larger number of time point observations in these experiments will allow for more reliable identification of regulated genes. In addition, continued improvements in computing technology will make it possible to apply the KM-algorithm to larger data sets in order to identify regulated genes. Finally, the novel model selection method that is applied as part of the KM-algorithm is applicable in many other fields where a large number of both observations and model parameters provide challenges.

Gene regulatory networks are only one example of a more general biological pathway. Other applications include the study of an organism's metabolome or proteome over time [34]. Analogous to gene expression applications, the KM-algorithm is general enough to be applied to any “omic” time series study, gained from any technology, whose purpose it is to identify regulated variables.

## Figures and Tables

**Table 1:** Percentages of correctly classified genes in the simulation whose results are depicted in Figure 1.

	Temporal variance		Regulation criterion	
Top	Regulated	Unregulated	Regulated	Unregulated
1%	(0/10) 0%	(970/990) 98.0%	(10/10) 100%	(980/990) 99.0%
5%	(2/50) 4.0%	(932/950) 98.1%	(19/50) 38%	(949/950) 99.9%
10%	(7/100) 7%	(887/900) 98.6%	(19/100) 19%	(899/900) 99.9%
20%	(7/200) 3.5%	(787/800) 98.4%	(20/200) 10%	(800/800) 100%

**Table 2:** The “goodness of rank” (GR) measure results for gene ranking obtained by applying the KM-algorithm to the complete and partitioned data sets with *n* = 2000 genes and *T* time point observations.

*T*	20	40	100
Complete data set	0.0421	0.1543	0.6434
Partitioning method	0.2012	0.2982	0.5349
